# V_2_O_5_ Nanospheres with Mixed Vanadium Valences as High Electrochemically Active Aqueous Zinc-Ion Battery Cathode

**DOI:** 10.1007/s40820-019-0256-2

**Published:** 2019-03-22

**Authors:** Fei Liu, Zixian Chen, Guozhao Fang, Ziqing Wang, Yangsheng Cai, Boya Tang, Jiang Zhou, Shuquan Liang

**Affiliations:** 1 School of Materials Science and Engineering, Central South University, Changsha, 410083 Hunan People’s Republic of China; 2 Key Laboratory of Electronic Packaging and Advanced Functional Materials of Hunan Province, Central South University, Changsha, 410083 Hunan People’s Republic of China

**Keywords:** V_2_O_5_, Mixed valences, Hollow sphere, Long-cycle-life, Aqueous zinc-ion battery

## Abstract

A V^4+^-V_2_O_5_ cathode with mixed vanadium valences was prepared via a novel synthetic method using VOOH as the precursor, and its zinc-ion storage performance was evaluated. The products are hollow spheres consisting of nanoflakes. The V^4+^-V_2_O_5_ cathode exhibits a prominent cycling performance, with a specific capacity of 140 mAh g^−1^ after 1000 cycles at 10 A g^−1^, and an excellent rate capability. The good electrochemical performance is attributed to the presence of V^4+^, which leads to higher electrochemical activity, lower polarization, faster ion diffusion, and higher electrical conductivity than V_2_O_5_ without V^4+^. This engineering strategy of valence state manipulation may pave the way for designing high-performance cathodes for elucidating advanced battery chemistry.

**Electronic supplementary material:**

The online version of this article (10.1007/s40820-019-0256-2) contains supplementary material, which is available to authorized users.

## Highlights

- Hollow V^4+^-V_2_O_5_ nanospheres are prepared by a novel and simple method using VOOH as the precursor.
- V^4+^-V_2_O_5_ with mixed vanadium valences is firstly constructed as an electrochemically active cathode for aqueous zinc-ion batteries.
- The V^4+^-V_2_O_5_ cathode exhibits a prominent cycling performance up to 1000 cycles and an excellent rate capability.

**Electronic supplementary material:**

The online version of this article (10.1007/s40820-019-0256-2) contains supplementary material, which is available to authorized users.

## Graphical abstract

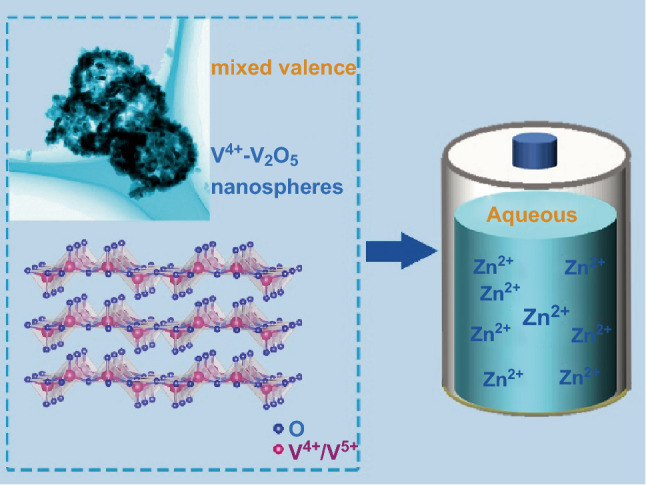

## Introduction

Although significant achievements have been made for the high energy density and long-cycle-life lithium-ion batteries practical applications, the limited lithium supply, high cost, and low safety impede their further development in large-scale energy storage [1–4], motivating us to find an alternative battery chemistry. As a result of the multiple electrons involved in redox reactions, aqueous multivalent ion battery systems possess higher energy density compared with battery systems and supercapacitors based on conventional aqueous alkali metal cations (e.g., Li^+^ and Na^+^) [5–8]. Recently, aqueous zinc-ion batteries (ZIBs) have captured much attention due to their low cost, high safety, and environmental friendliness [9–11]. Furthermore, the use of non-toxic and safe aqueous electrolytes with high ionic conductivity makes the aqueous ZIBs a promising battery chemistry for grid-scale applications [12]. So far, many cathode materials have been developed for aqueous ZIBs, but they still suffer from poor electrochemical performance, such as drastic capacity fading and inferior rate capability for manganese-based cathodes [13–19] and low capacity for Prussian blue analogs [20, 21].

Recently, vanadium-based compounds are receiving intensive research interest as cathode materials for aqueous ZIBs, owing to the multiple oxidation states of vanadium and its abundant supply [10, 22–24]. Since Nazar’s group developed Zn_0.25_V_2_O_5_nH_2_O as a cathode with a high energy density of 250 Wh kg^−1^ and good cycle stability up to 1000 cycles for aqueous ZIBs [25], a series of compounds such as Ca_0.25_V_2_O_5_·nH_2_O [26], K_0.25_V_2_O_5_ [27], Na_2_V_6_O_16_.1.63H_2_O [28–30], Na_0.33_V_2_O_5_ [31], NH_4_V_4_O_10_ [32], and Mg_*x*_V_2_O_5_·nH_2_O [33] have been explored. In fact, vanadium is in a mixed valence state in these materials due to the insertion of guest ions. However, the introduction of guest ions may increase the molar mass and decrease the specific capacity to some extent. The pure phase of V_2_O_5_ has been demonstrated with poor performance as a cathode for aqueous ZIBs, due to its poor electronic and ionic conductivities [22, 34].

Inspired by the reported mixed valence states of vanadium oxides with enhanced electrochemical performance for energy application [35–37], we have, for the first time, prepared V^4+^-V_2_O_5_ hollow nanospheres by a novel synthetic method for application in zinc-ion storage cathodes. It is worth noting that V^4+^-V_2_O_5_ possesses higher electrochemical activity, lower polarization, faster ion transport, and better electrical conductivity than V_2_O_5_. As expected, V^4+^-V_2_O_5_ exhibits superior electrochemical performances as a cathode for aqueous ZIBs, with high capacity, excellent rate capability, and long-term cyclic life up to 1000 cycles. Moreover, the presented ZIB system using 2 M ZnSO_4_ aqueous solution as electrolyte is cost-effective and its electrochemical properties are excellent, which makes it practical for large-scale applications.

## Experimental Section

### Materials Synthesis

VOOH is synthesized based on the method reported by Xie’ s group [38]. Firstly, 2 mmol NH_4_VO_3_ was dissolved into a beaker containing 45 mL of deionized water and was stirred vigorously for 10 min. Secondly, 5 mL of 1 M HCl solution was injected into the beaker until the turbid liquid turned into a yellow transparent solution, at a rate of 1 mL per minute. Thirdly, 5 mL of N_2_H_4_·3H_2_O, employed as a strong reducing agent, were added to the previously prepared solution while stirring continuously for 30 min. Then, the obtained V(OH)_2_NH_2_ brown turbid fluid was transferred to a Teflon-lined stainless-steel autoclave and kept in an electrical oven at 120 °C for 8 h. The precursor VOOH was prepared via suction filtration and was dried at 50 °C in vacuum. V^4+^-V_2_O_5_ was obtained by annealing the precursor in air atmosphere for 6 h at 250 °C with a heating rate of 2 °C min^−1^. V_2_O_5_ with pure pentavalent vanadium can be obtained at temperatures above 300 °C.

### Materials Characterization

A combined differential scanning calorimetry (DSC)/thermogravimetric analysis (TG) instrument (Netzsch STA449 C, Germany) was used to study the evolution of VOOH in air at a heating ramp rate of 10 °C min^−1^. The phase composition of the as-prepared compounds was analyzed by X-ray power diffraction (XRD) patterns detected with a Rigaku D/MAX-2500 diffractometer (Cu Kα). The phase transformation process was monitored by high-temperature dynamic XRD (Rigaku SmartLab, Cu Kα), with the temperature increasing from 50 to 350 °C at a heating rate of 10 °C min^−1^ and was kept warm for 10 min. The morphology features were obtained by scanning electron microscopy (SEM, Quanta FEG 250). Transmission electron microscopy (TEM), high-resolution transmission electron microscopy (HRTEM), selected area electron diffraction (SAED), and TEM/energy-dispersive spectroscopy (TEM–EDS) mapping were carried out on the transmission electron microscope (Tecnai G2 F20). X-ray photoelectron spectroscopy (XPS) measurements taken on a spectrometer (Escalab 250xi, Thermo Scientific) explain the valence state of the elements in the product.

### Electrode Fabrication and Electrochemical Measurements

The electrochemical properties of the as-prepared compounds were tested via CR2016 coin cells. The cathode electrodes were fabricated by coating a stainless-steel wire mesh with ropy slurry and drying it in a vacuum oven at 80 °C for 12 h. The slurry was prepared by mixing active material (70 wt%), acetylene black (20 wt%), polyvinylidene fluoride binder (10 wt%), and *N*-methyl-2-pyrrolidone. A metal zinc plate was utilized as anode, and a 2 M ZnSO_4_ aqueous solution was employed as electrolyte. The mass loading of active materials on the electrode was approximately 1.5 mg cm^−2^. The electrochemical behavior was evaluated at voltages between 0.4 and 1.4 V. Galvanostatic charge/discharge tests were carried out with a multichannel battery testing system (LAND CT2001A). Cyclic voltammetry (CV) tests were carried out with the CHI 660e electrochemical station. Electrochemical impedance spectrometry (EIS) measurements were performed in the frequency range of 100 kHz to 10 mHz on a ZAHNER-IM6ex electrochemical workstation (Kronach, Germany). All the electrochemical measurements were carried out at a controlled room temperature of 28 °C.

## Results and Discussion

**Fig. 1:**
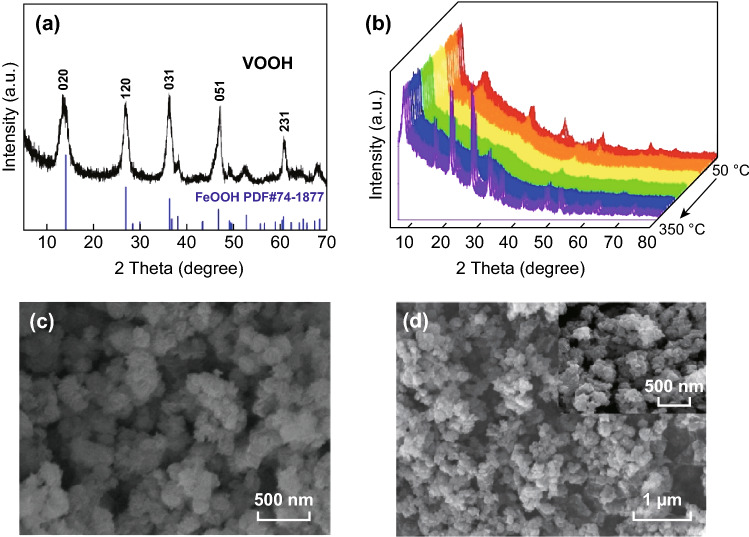
**a** XRD pattern of VOOH, **b** dynamic high-temperature XRD image of VOOH, SEM images of **c** VOOH and **d** V^4+^-V_2_O_5_

**Fig. 2:**
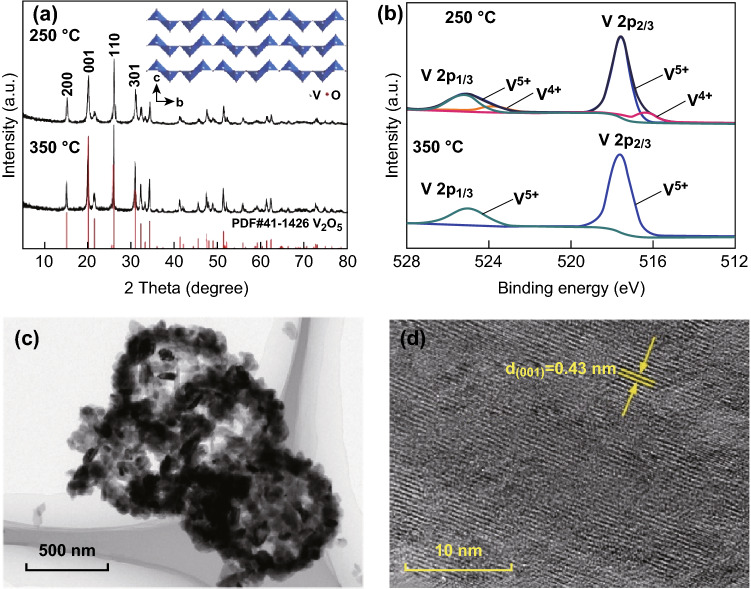
**a** XRD pattern and crystal structure of V^4+^-V_2_O_5_. **b** XPS spectra for V 2p of V^4+^-V_2_O_5_ and V_2_O_5_. **c** TEM image and **d** HRTEM image of V^4+^-V_2_O_5_

The XRD peaks of the synthesized precursor (Fig. 1a) are consistent with the patterns of the previously reported VOOH [39], which has a structure analogous to orthorhombic FeOOH (PDF#74-1877). However, this compound is not suitable for storage of Zn^2+^ ions, as indicated in Fig. S1. It is reported that V_2_O_5_ is a promising cathode for aqueous ZIBs [22, 34, 40, 41]. Temperature-controlled in situ XRD (Fig. 1b) was conducted to monitor the phase transformation process of VOOH. As the temperature increases, the substance initially goes through an amorphous state with several broad and weak diffraction peaks, and then transforms into the stable vanadium pentoxide phase. The TG–DSC result for VOOH (Fig. S2) is consistent with the dynamic high-temperature XRD data. Thus, the VOOH was sintered in air with temperatures increasing from 250 to 350 °C for 6 h at a heating rate of 2 °C min^−1^ to obtain the different vanadium valences of V_2_O_5_. The SEM image (Fig. 1c) demonstrates that the precursor VOOH has a spherical morphology. The V_2_O_5_ products obtained at different temperatures have flaky morphologies similar to that of VOOH (Fig. 1d and S3). As seen in Fig. 2a, the XRD patterns of the samples prepared at 250 and 350 °C can be assigned to V_2_O_5_ with an orthorhombic structure (PDF#41-1426). The crystal structure of V_2_O_5_ (inset Fig. 2a) with a layered framework can provide enough space for insertion/extraction of Zn ions. The XPS analysis was used to investigate the chemical oxidation state of vanadium in V_2_O_5_ obtained at different temperatures (Fig. 2b). The binding energies of XPS spectra were calibrated using C 1 s = 284.5 eV as a reference. The V 2p_3/2_ and V 2p_1/2_ peaks of V_2_O_5_ obtained at 350 °C were located at 517.6 and 525 eV, confirming that the vanadium element is in the pentavalent state (V^5+^) due to the high-temperature calcination [42]. However, the V 2p peaks of V_2_O_5_ obtained at 250 °C present two extra peaks at 516.4 and 523.8 eV, which correspond to the tetravalent vanadium (V^4+^) [43]. This implies that the vanadium in V_2_O_5_ obtained at 250 °C is not completely oxidized during the low-temperature calcination. The relative calculated molar ratio of V^5+^ to V^4+^ is 4.74:1. Hereafter, we refer to the samples prepared at 250 and 350 °C as V^4+^-V_2_O_5_ and V_2_O_5_, respectively. The TEM image of V^4+^-V_2_O_5_ (Fig. 2c) further confirms that the nanoparticles are hollow spheres consisting of nanoflakes. This unique nanostructure can provide more active sites and increase the ion diffusion ability [44]. A typical interplanar spacing of 0.43 nm corresponds to the (001) planes of V_2_O_5_ (Fig. 2d).

**Fig. 3:**
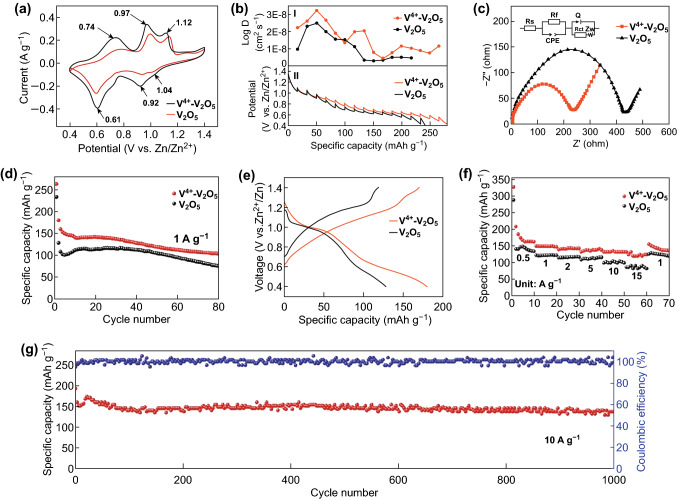
**a** The third cycle of the CV curves of the V^4+^-V_2_O_5_ and V_2_O_5_ electrodes at a scan rate of 0.1 mV s^−1^ in the voltage range 0.4–1.4 V (vs. Zn/Zn^2+^). **b** GITT curves (I) and calculated diffusion coefficients (II) of V^4+^-V_2_O_5_ and V_2_O_5_ electrodes. **c** Nyquist plots of V^4+^-V_2_O_5_ and V_2_O_5_ before cycling. **d** Cycling performances of V^4+^-V_2_O_5_ and V_2_O_5_ electrodes at a discharge current density of 1 A g^−1^. **e** Discharge–charge voltage profiles of V^4+^-V_2_O_5_ and V_2_O_5_ electrodes in the second cycle at 1 A g^−1^. **f** Rate capability of V^4+^-V_2_O_5_ and V_2_O_5_ electrodes at various current rates. **g** Long-term cycling performance of the Zn/V^4+^-V_2_O_5_ battery at 10 A g^−1^

Figure 3a displays the CV curves of V^4+^-V_2_O_5_ and V_2_O_5_ at the scan rate of 0.1 mV s^−1^ and voltage range of 0.4–1.4 V (vs. Zn/Zn^2+^). Both samples exhibit three main redox couples, such as 0.61/0.74, 0.92/0.97, and 1.04/1.12 for V^4+^-V_2_O_5_. It should be mentioned that the gap between the redox peaks of V^4+^-V_2_O_5_ is smaller than that of V_2_O_5_, indicating the lower polarization of V^4+^-V_2_O_5_ [45, 46]. Furthermore, the peak current densities of V^4+^-V_2_O_5_ are much stronger than those of V_2_O_5_, suggesting that the former has a higher electrochemical reactivity and higher capacity than the latter [47]. To further demonstrate the advantages of V^4+^-V_2_O_5_, the zinc diffusion coefficient was measured via galvanostatic intermittence titration techniques (GITT) and calculated according to Eq. (1) [48]:

1 $$ D = \frac{{4L^{2} }}{\pi \tau }\left( {\frac{{\Delta E_{\text{s}} }}{{\Delta E_{\text{t}} }}} \right)^{2} $$

where *τ* represents relaxation time (*s*) and *L* corresponds to Zn^2+^ diffusion length (cm). $\Delta E_{\text{s}}$ is the steady-state potential change (*V*) by the current pulse. $\Delta E_{\text{t}}$ is the voltage change (*V*) during the constant current pulse (eliminating the voltage changes after relaxation time). In GITT, current pulse of 100 mA g^−1^ was applied for 600 s while the followed relaxation time is 1800 s. The GITT measurement continues until the cut off potential is reached. The GITT curves and specific zinc diffusion coefficients (*D*_Zn_^2+^) of V^4+^-V_2_O_5_ and V_2_O_5_ are calculated and summarized in Fig. 3b. The *D*_Zn_^2+^ values in V^4+^-V_2_O_5_ range from 4.31E^−9^ to 3.24E^−8^, while those in V_2_O_5_ range from 2.82E^−9^ to 2.50E^−8^. It is obvious that the *D*_Zn_^2+^ in V_2_O_5_ is much lower than that in V^4+^-V_2_O_5_ (Fig. 3b). We also performed EIS to analyze the impedance difference between V^4+^-V_2_O_5_ and V_2_O_5_, as displayed in Fig. 3c. Both batteries were tested after being assembled and allowed to sit for 4 h, with an open-circuit voltage of approximately 1.28 V. The impedance patterns exhibit a semicircle in the high-frequency range and a sloped line in the low-frequency range. The formation of the semicircle is attributed to the solid electrolyte interface (SEI) film and the charge-transfer reaction at the electrode/electrolyte interface [49–51]. The inset in Fig. 3c shows the equivalent circuit model for the impedance spectra, where *R*_s_ represents the combination of the electrolyte resistance and ohmic resistances of the cell components and *R*_f_ and *R*_ct_ represent the resistance of the SEI films and the charge-transfer resistance of the electrochemical reaction, respectively. CPE, Q, and *Z*_w_ are the surface-passivating layer capacitance, double-layer capacitance, and diffusion-controlled Warburg impedance, respectively. The fitting parameters are listed in Table S1. It is evident that the charge-transfer resistance value of the V^4+^-V_2_O_5_ electrode is much lower than that of the V_2_O_5_ electrode. The *R*_ct_ of the electrochemical reaction for V^4+^-V_2_O_5_ and V_2_O_5_ are 203.5 and 377.3 Ω, respectively. The above results suggest that the V^4+^-V_2_O_5_ with the presence of V^4+^ possesses higher electrochemical activity, faster ion diffusion capability, and better electrical conductivity, which are expected to lead to better electrochemical performances than those of V_2_O_5_.

The electrochemical performance of V^4+^-V_2_O_5_ and V_2_O_5_ as cathodes in aqueous ZIBs is evaluated. V^4+^-V_2_O_5_ and V_2_O_5_ exhibit an initial specific capacity of 262.1 and 249.6 mAh g^−1^ at a current density of 1 A g^−1^, respectively (Fig. 3d). After 80 cycles, the specific capacities of both samples decrease rapidly, while V^4+^-V_2_O_5_ displays a better cyclic stability. The reason for the rapid decrease in specific capacity in the initial cycles will be discussed later. The selected discharge/charge voltage profile of V^4+^-V_2_O_5_ has three platforms at approximately 1.1, 1.0, and 0.6 V (Fig. 3e), which indicate a much more obvious Zn^2+^ insertion/extraction behavior than in V_2_O_5_. The V^4+^-V_2_O_5_ also exhibits superior rate capability with the average specific discharge capacities of 188.7, 149.9, 143, 138.31, 133, and 124.93 mAh g^−1^ at current densities of 0.5, 1, 2, 5, 10, and 15 A g^−1^, respectively (Fig. 3f). However, the V_2_O_5_ cathode exhibits a low capacity of 87.5 mAh g^−1^ at 15 A g^−1^. Furthermore, the V^4+^-V_2_O_5_ cathode exhibits a long-term cycling performance at a high current density of 10 A g^−1^, at which a high specific capacity of 140 mAh g^−1^ can be maintained after 1000 cycles (Fig. 3g).

As shown in Fig. S4, the V_2_O_5_ samples obtained at different temperatures exhibit similar electrochemical properties, indicating that the crystallinity may have a slight impact on the electrochemical performance of the as-prepared samples. The SEM images of V^4+^-V_2_O_5_ and V_2_O_5_ (Fig. 1d and S3) show that both have spherical morphology and are composed of nanosheets with similar size and shape, so the effect of morphology on the electrochemical performance difference could be ignored. As a result, the improved electrochemical performance of V^4+^-V_2_O_5_ compared to V_2_O_5_ may be due to the mixed valence states. It is known that introducing mixed valences of metal ions in electrode materials has appreciable impacts on their electrochemical reactions [35, 36, 52]. The presence of such defects at the electrode interface could not only increase the effective contact area between electrode and electrolyte [53], but also behave like a protective coating layer to maintain the morphology stability of the electrode [36, 37].

**Fig. 4:**
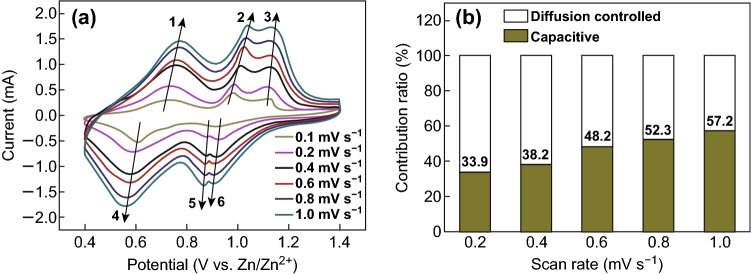
**a** CV curves at different scan rates. **b** Bar chart showing the percentage of pseudocapacitive contribution of V^4+^-V_2_O_5_ at different scan rates

The electrochemical reaction kinetics of V^4+^-V_2_O_5_ are further investigated by CV curves at different scan rates from 0.1 to 1 mV s^−1^ (Fig. 4a). The relationship between the current *i* (mA) and the scan rate *v* (mV s^−1^) is shown in Eqs. (2) and (3) [54]:

2 $$ i = av^{\text{b}} $$

3 $$ \log \left( i \right) = b\log \left( v \right) + \log \left( a \right) $$

where *a* and *b* are variable parameters. The calculated *b* values of the six redox peaks are 0.68, 0.64, 0.71, 0.56, 0.83, and 0.77, indicating that the partial intercalation pseudocapacitance contributes to the capacity of V^4+^-V_2_O_5_, thus leading to the fast diffusion of Zn^2+^. The intercalation pseudocapacitance contribution ratio could be calculated by Eqs. (4) and (5) [55]:

4 $$ i = k_{1} v + k_{2} v^{{\frac{1}{2}}} $$

5 $$ \frac{i}{{v^{{\frac{1}{2}}} }} = k_{1} v^{{\frac{1}{2}}} + k_{2} . $$

The area ratio of the shaded region in Fig. 4b illustrates that the intercalation pseudocapacitance contribution ratio of V^4+^-V_2_O_5_ raises from 33.9% to 57.2% as the scan rate increases from 0.2 to 1 mV s^−1^.

**Fig. 5:**
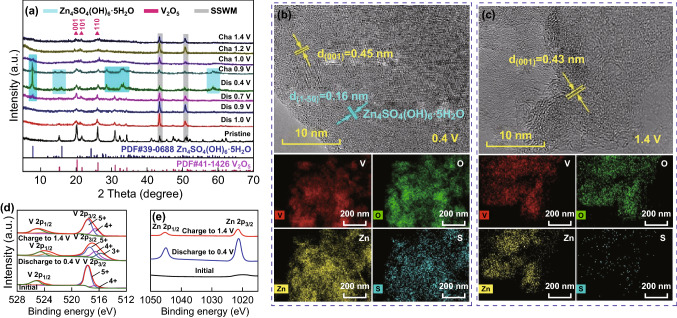
Analysis of the Zn storage mechanism of the V^4+^-V_2_O_5_ electrode. **a** Ex situ XRD patterns of V^4+^-V_2_O_5_ electrodes discharged or charged to different voltage states at the current density of 100 mAg^−1^, HRTEM, and TEM-EDS mapping images of the electrodes **b** discharged to 0.4 V and **c** charged to 1.4 V, ex situ high-resolution XPS spectra of **d** V 2p and **e** Zn 2p at the fully discharged/charged state

An ex situ XRD technique was employed to explore the structural changes of V^4+^-V_2_O_5_ during the discharge/charge process (Fig. 5a). The diffraction peaks at 20.262°, 21.711°, and 26.126°, corresponding to the (001), (101), and (110) lattice planes, respectively, shift to slightly lower angles during the discharging process, suggesting that interlayer spacing of the V^4+^-V_2_O_5_ increases due to the insertion of Zn^2+^. When charging to a higher voltage, the characteristic peaks become broader, which could be explained by the fact that the de-intercalation of Zn^2+^ ions from the layered structure results in lattice distortions. It should be noted that a new phase of Zn_4_SO_4_(OH)_6_·5H_2_O (PDF#39-0688) is generated during the discharge process. When the electrode was charged from 0.4 to 1.4 V, the Zn_4_SO_4_(OH)_6_·5H_2_O phase gradually disappeared. The appearance and disappearance of the Zn_4_SO_4_(OH)_6_·5H_2_O phase is similar to the mechanism of zinc/sodium vanadate batteries reported by Niu’s, who illustrates the co-insertion of Zn^2+^/H^+^ contributing to the high capacity [24]. The ex situ XRD patterns of V^4+^-V_2_O_5_ electrodes discharged or charged to different voltages at 100 mA g^−1^ in the second cycle (Fig. S5) demonstrate a similar zinc storage mechanism. Furthermore, the ex situ XRD patterns of the V^4+^-V_2_O_5_ electrode charged to 1.4 V at different cycles (Fig. S6) are similar to those of the initial sample, demonstrating that the insertion/extraction mechanism of Zn^2+^ ions in V^4+^-V_2_O_5_ electrode is reversible in the following cycles. It can be seen that the location of the diffraction peaks corresponding to the (001), (101), and (110) lattice planes is consistent with that in the initial sample, indicating that the sample is still a layered structure of V_2_O_5_ in the charge–discharge process. SEM images of the V^4+^-V_2_O_5_ electrode with different magnifications and at different discharge/charge states are shown in Fig. S7. It can be seen that the morphology of V^4+^-V_2_O_5_ during cycling is not as regular as in the original state, which may further explain the poor cycling stability at low current densities.

The structural changes of V^4+^-V_2_O_5_ are further evaluated by the ex situ HRTEM images of the electrodes discharged to 0.4 V and charged to 1.4 V (Fig. 5b, c). The interplanar spacing of (001) is 0.45 nm at the discharged state of 0.4 V, while it is 0.43 nm at the charged state of 1.4 V. Compared with the original state, such data confirm the insertion/extraction behavior of Zn^2+^ ions in the V^4+^-V_2_O_5_ electrode. The interplanar spacing of 0.16 nm at 0.4 V matches well that of the Zn_4_SO_4_(OH)_6_·5H_2_O (PDF#39-0688) state. Figure S8 displays the SAED images of V^4+^-V_2_O_5_ at different states. According to the extinction law of orthogonal crystal systems, the (001) crystal faces cannot be seen in the SAED images. We found (002) crystal faces with half the interplanar spacing of the (001) crystal faces (Fig. S8a). When discharged to 0.4 V, several obscure rings were observed, which may be classified as the new phase of Zn_4_SO_4_(OH)_6_·5H_2_O (Fig. S8b). When charged to 1.4 V, the substance appears to be a single and amorphous mixture (Fig. S8c), which is consistent with the XRD result.

The rapid decrease in the specific capacity in the initial stage may be due to the fact that some zinc ions located at the “dead Zn^2+^ sites” cannot be extracted from the V_2_O_5_ lattice in the charge process [56, 57], which can be further revealed in the TEM-EDS mapping images of the electrode discharged/charged to 0.4 V/1.4 V (Fig. 5b, c). It is reported that the zinc ions that fail to exit from the host structure during the charging process may act as layer pillars, making the structure of V_2_O_5_ more stable [41]. We also concentrated on the valence state changes of vanadium, as presented in Fig. 5d. When discharged to 0.4 V, the V 2p_3/2_ peaks separated into three peaks located at 517.3, 516.4, and 515.5 V, which correspond to V^5+^, V^4+^, and V^3+^, respectively, and the V 2p_1/2_ peaks located at 524.5, 523.6, and 522.6 V also correspond to V^5+^, V^4+^, and V^3+^, respectively, indicating the reduction in vanadium accomplished by the insertion of Zn^2+^. The vanadium is further oxidized during the charging process. Note that the portion of V^4+^ is higher than that of its original state, which may be due to the incomplete extraction of Zn^2+^. This phenomenon is consistent with the high-resolution Zn 2p XPS spectra (Fig. 5e).

**Fig. 6:**
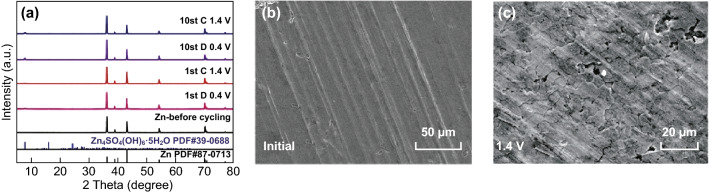
**a** XRD patterns of Zn anodes from Zn/V^4+^-V_2_O_5_ batteries at different states. SEM images of Zn anodes from Zn/V^4+^-V_2_O_5_ batteries at different states: **b** initial and **c** charged to 1.4 V

Investigations on Zn anodes were also carried out. Figure 6a presents the XRD patterns of the Zn anodes from Zn/V^4+^-V_2_O_5_ batteries at different states. There is no obvious Zn oxidation during the charging and discharging process. It can be observed that only a small amount of Zn_4_SO_4_(OH)_6_·5H_2_O is formed after 10 cycles. Compared with the initial metallic Zn plate (Fig. 6b), the SEM image of the Zn anode after one cycle demonstrates that the Zn plate still exhibits a dense and dendrite-free morphology (Fig. 6c).

## Conclusion

In summary, we have successfully synthesized V^4+^-V_2_O_5_ and V_2_O_5_ hollow spheres with different oxidation states of vanadium, by controlling the sintering process of the VOOH precursor. With the CV, GITT, and EIS techniques, we demonstrated that V^4+^-V_2_O_5_ with mixed vanadium valences exhibits higher electrochemical activity, lower polarization, faster ion diffusion capability, and higher electrical conductivity than V_2_O_5_. As expected, the V^4+^-V_2_O_5_ cathode exhibits excellent Zn^2+^ storage performances. For instance, it can maintain a high specific capacity of 140 mAh g^−1^ after 1000 cycles at 10 A g^−1^ and presents outstanding rate capability. The extra tetravalent vanadium ions could increase the electronic and ionic conductivities. The results suggest that V^4+^-V_2_O_5_ is a promising cathode for aqueous ZIBs.

## Electronic supplementary material

Below is the link to the electronic supplementary material.

Supplementary material 1 (PDF 796 kb)
