# Supplementary material for: V2O5 Nanospheres with Mixed Vanadium Valences as High Electrochemically Active Aqueous Zinc-Ion Battery Cathode
Source: Nanomicro Lett. 2019 Mar 22;11:25. doi: 10.1007/s40820-019-0256-2 (PMC7770672; doi:10.1007/s40820-019-0256-2)
Supplement: Supplementary file 1 — Supplementary material 1 (PDF 796 kb) [file 40820_2019_256_MOESM1_ESM.pdf]

Supporting Information for

V_2O_5 Nanospheres with Mixed Vanadium Valences as High Electrochemically Active Aqueous Zinc-ion Battery Cathode

Fei Liu[1], Zixian Chen[1], Guozhao Fang[1], Ziqing Wang[1], Yangsheng Cai[1], Boya Tang[1], Jiang Zhou[1, 2, *], Shuquan Liang[1, 2, *]

[1]School of Materials Science and Engineering, Central South University, Changsha, Hunan 410083, People's Republic of China

[2]Key Laboratory of Electronic Packaging and Advanced Functional Materials of Hunan Province, Central South University, Changsha, Hunan 410083, People's Republic of China

*Corresponding authors. E-mail: zhou_jiang@csu.edu.cn (Jiang Zhou); lsq@csu.edu.cn (Shuquan Liang)

Supplementary Figures and Table

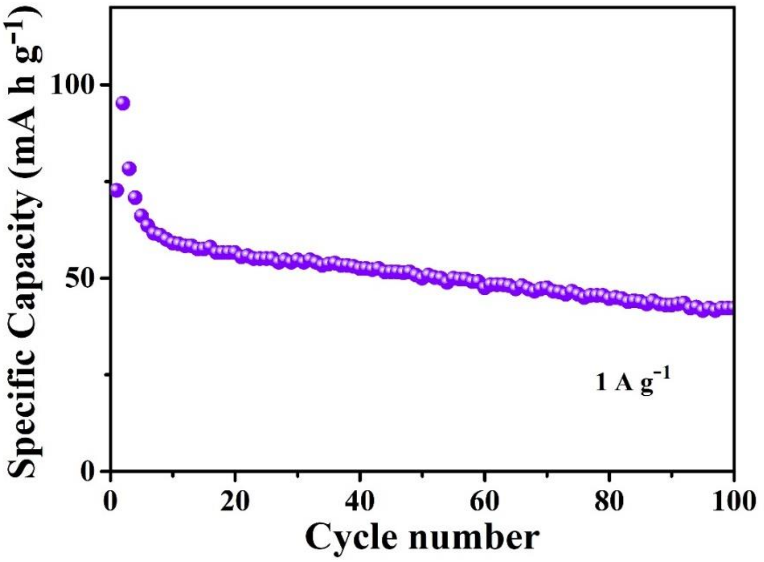

Fig. S1 Cycle performance at 1 A g^{-1} of VOOH electrode

When the current density is 1 A g^{-1}, the first discharge specific capacity of the Zn/VOOH coin cell is 75 mAh g^{-1}. After 100 cycles, the specific capacity drops to 50 mAh g^{-1}. Obviously, VOOH is not suitable to be used as the cathode material for ZIBs.

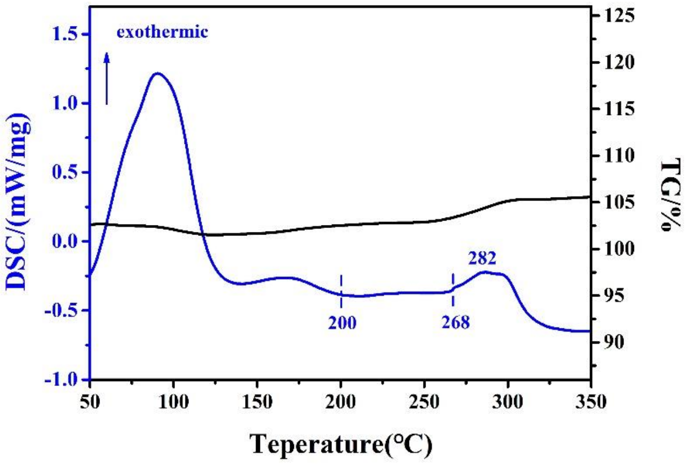

Fig. S2 TG and DSC results of VOOH dried at 50 ℃. The temperature ramp rate was set to 10 ℃ min^{-1}

Figure S2 shows the TG-DSC results of the VOOH and we can divide the heating process into three parts for analysis. First, before heating to 200 °C, V^{3+} is slowly oxidized to V^{4+} in air. Second, the substance is a mixed valence state of V^{4+} and V^{5+} within the temperature range of 200-270 °C as indicated in **Fig. S2.** Third, after heating to 282 °C, the substance in mixed valence state of V^{4+} and V^{5+} is converted into V_2O_5 in pure pentavalent gradually. During the oxidation process, the mass is slightly increased due to the absorption of oxygen in air. And then the quality is unchanged, implying that the vanadium in substance is completely oxidized, which corresponds to the stable vanadium pentoxide phase in the temperature-controlled *in-situ* XRD (**Fig. 1b**).

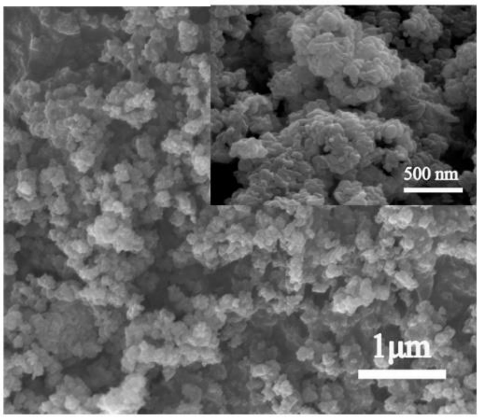

Fig. S3 SEM image of V_2O_5

Table S1 EIS primary simulation parameters of as-prepared V^{4+}-V_2O_5 and V_2O_5

Samples	$R_s(\Omega)$	$R_{ct}(\Omega)$
V^{4+}-V_2O_5	1.212	203.5
V_2O_5	2.456	377.3

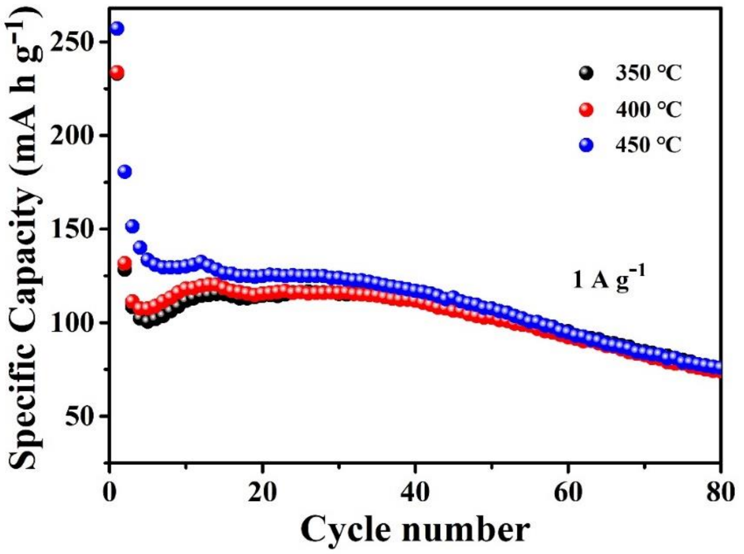

Fig. S4 Cycling performance of samples obtained at 350 °C, 400 °C, and 450 °C at the discharge current density of 1 A g^{-1}

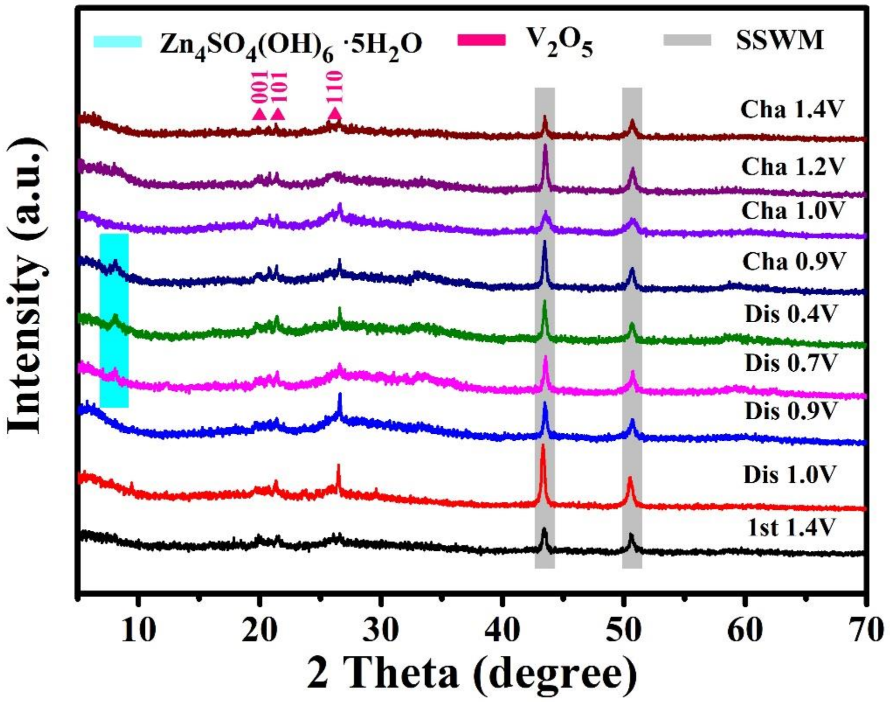

Fig. S5 Ex-situ XRD patterns of V^{4+}-V_2O_5 electrodes discharged or charged to different voltage states at the current density of 100 mA g^{-1} in the second cycle

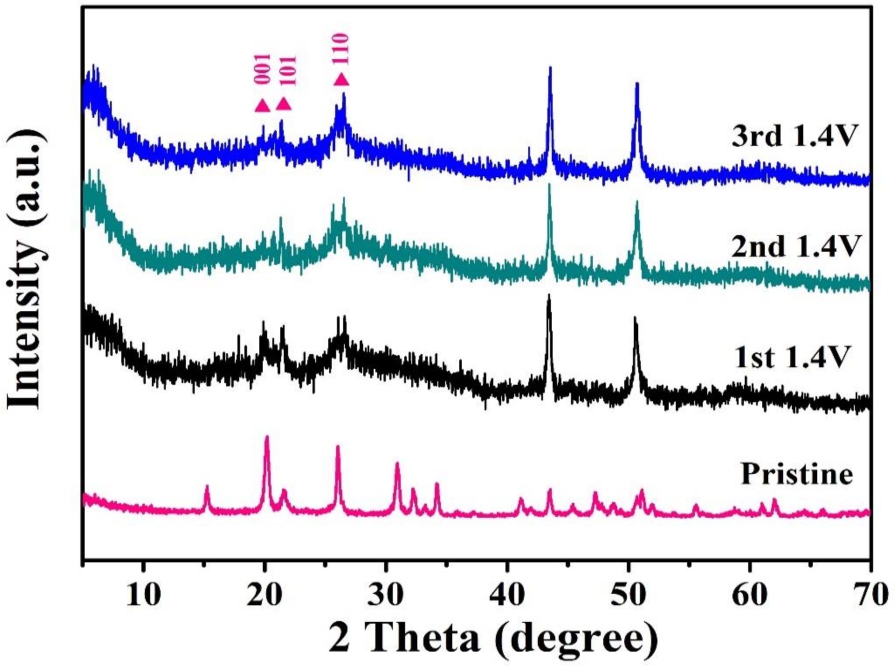

Fig. S6 Ex-situ XRD patterns of V^{4+}-V_2O_5 electrodes charged to 1.4 V at different cycles

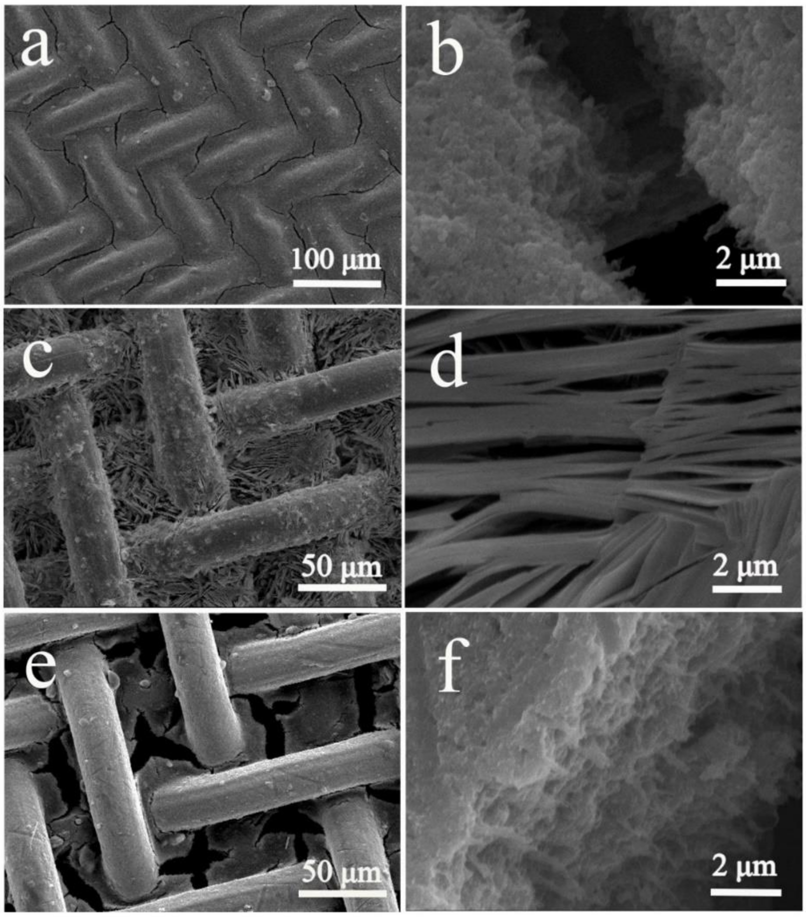

Fig. S7 SEM images with different magnifications of V^{4+}-V_2O_5 electrode film at **a**, **b** original state. **c, d** discharge to 0.4 V. **e, f** charge to 1.4 V

The scanning electron microscopy (SEM) images of V^{4+}-V_2O_5 electrodes at different states are shown in **Fig. S7**. The SEM image of initial V^{4+}-V_2O_5 electrode is presented in **Fig. S7a**. Furthermore, the SEM images of the surface and cross-section of electrode (**Fig. S7b**) also reflect that the surface and internal structure of the mixture are relatively loose, which is beneficial to the Zn^{2+} diffusion. When discharged to 0.4 V, it can be seen that the morphology of the material on the surface of the electrode film changed a lot. There are many layered flakes emerged compared to the initial state **(Fig. S7c, d)**, these layered flakes vanished during charging process (**Fig. S7e**). The highly reversible morphologic transformation during the discharge/charge process is well consistent with the evolution observed in ex-situ XRD patterns **(Fig. 5)**, which corresponding to the formation/disappearance of $Zn_4SO_4(OH)_6$•$5H_2O$ phase. What's more, such phenomenon has been reported in some previous reports[S1-S3]. The flakes at discharge states may be due to the formation of $Zn_4SO_4(OH)_6$•$5H_2O$. When charged to 1.4 V, it is obviously that the morphology of V^{4+}-V_2O_5 electrode film is not as regular as the original state, it becomes a little messy **(Fig. S7e, f)**, which may further explain the phenomenon that the cycling stability in low current density is not very good.

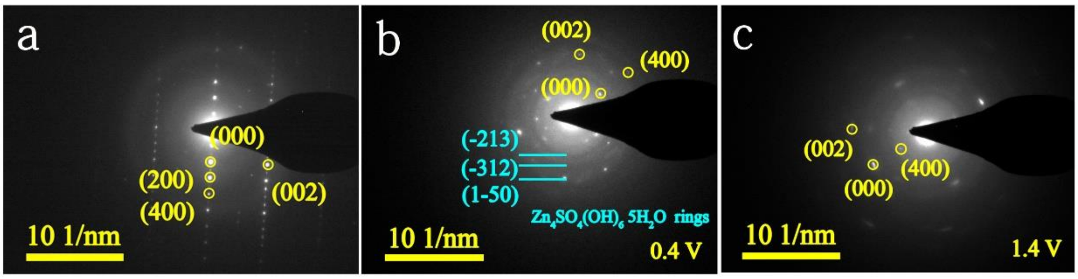

Fig. S8 SAED images of V^{4+}-V_2O_5 **a** powder **b** discharged to 0.4 V and **c** charged to 1.4 V

Supplementary References

[S1]M. Chamoun, W.R. Brant, C.-W. Tai, G. Karlsson, D. Noréus, Rechargeability of aqueous sulfate Zn/MnO_2 batteries enhanced by accessible Mn^{2+} ions. Energy Storage Mater. **15**, 351-360 (2018). https://doi.org/10.1016/j.ensm.2018.06.019

[S2]J. Huang, Z. Wang, M. Hou, X. Dong, Y. Liu, Y. Wang, Y. Xia, Polyaniline-intercalated manganese dioxide nanolayers as a high-performance cathode material for an aqueous zinc-ion battery. Nat. Commun. **9**(1), 2906 (2018). https://doi.org/10.1038/s41467-018-04949-4

[S3]C. Zhu, G. Fang, J. Zhou, J. Guo, Z. Wang, C. Wang, J. Li, Y. Tang, S. Liang, Binder-free stainless steel@Mn_3O_4 nanoflower composite: a high-activity aqueous zinc-ion battery cathode with high-capacity and long-cycle-life. J. Mater. Chem. A **6**(20), 9677-9683 (2018). https://doi.org/10.1039/c8ta01198b
